# Genewise detection of variants in *MEFV* gene using nanopore sequencing

**DOI:** 10.3389/fgene.2024.1493295

**Published:** 2024-11-29

**Authors:** Lilit Ghukasyan, Gisane Khachatryan, Tamara Sirunyan, Arpine Minasyan, Siras Hakobyan, Andranik Chavushyan, Varduhi Hayrapetyan, Hovsep Ghazaryan, Gevorg Martirosyan, Gohar Mkrtchyan, Valentina Vardanyan, Vahan Mukuchyan, Ashot Davidyants, Roksana Zakharyan, Arsen Arakelyan

**Affiliations:** ^1^ Laboratory of Human Genetics, Institute of Molecular Biology NAS RA, Yerevan, Armenia; ^2^ Department of Bioengineering, Bioinformatics and Molecular Biology, Institute of Biomedicine and Pharmacy, Russian-Armenian University, Yerevan, Armenia; ^3^ Research Group of Bioinformatics, Institute of Molecular Biology NAS RA, Yerevan, Armenia; ^4^ Armenian Bioinformatics Institute, Yerevan, Armenia; ^5^ Department of Rheumatology, Yerevan State Medical University after Mkhitar Heratsi (YSMU), Yerevan, Armenia; ^6^ Department of Rheumatology, “Mikaelyan” Institute of Surgery, Yerevan, Armenia; ^7^ Department of Internal Medicine and Rheumatology, Nairi Medical Center, Yerevan, Armenia; ^8^ “Gisane” LLC, Davidyants Laboratories, Yerevan, Armenia

**Keywords:** familial mediterranean fever, FMF, MEFV, nanopore sequencing, variant detection, genetic testing

## Abstract

Familial Mediterranean Fever (FMF) is a genetic disorder with complex inheritance patterns and genotype-phenotype associations, and it is highly prevalent in Armenia. FMF typically follows an autosomal recessive inheritance pattern (OMIM: 249100), though it can occasionally display a rare dominant inheritance pattern with variable penetrance (OMIM։134610). The disease is caused by mutations in the *MEFV* gene, which encodes the pyrin protein. While the 26 most prevalent mutations account for nearly 99% of all FMF cases, more than 60 pathogenic mutations have been identified. In this study, we aimed to develop an affordable nanopore sequencing method for full-length *MEFV* gene mutation detection to aid in the diagnosis and screening of FMF. We employed a multiplex amplicon sequencing approach, allowing for the processing of up to 12 samples on both Flow cells and Flongle flow cells. The results demonstrated near-complete concordance between nanopore variant calling and qPCR genotypes. Moreover, nanopore sequencing identified additional variants, which were confirmed by whole exome sequencing. Additionally, intronic and UTR variants were detected. Our findings demonstrate the feasibility of full-gene nanopore sequencing for detecting FMF-associated pathogenic variants. The method is cost-effective, with costs comparable to those of the qPCR test, making it particularly suitable for settings with limited laboratory infrastructure. Further clinical validation using larger sample cohorts will be necessary.

## 1 Introduction

Familial Mediterranean fever (FMF) is a genetic autoinflammatory disease characterized by mutations within the *MEFV* (MEditerranean FeVer) gene that encodes the pyrin protein (French FMF Consortium, 1997; The International FMF Consortium, 1997; Papin et al., 2000).

Despite being considered a rare disease worldwide with an estimate of over 200,000 affected people (Gallego et al., 2023), it is common in populations of Mediterranean descent, including Armenians, Turks, Jews, Greeks, and Italians with a prevalence ranging from 10 to 1,500 per 100,000 population (Cerrito et al., 2015; Alparslan et al., 2020). Moreover, FMF prevalence (13–20/per 100,000) is notable in Japan (Migita et al., 2016), and recently it has also been reported in China (Wu et al., 2018).

As of today, 399 variants in the *MEFV* gene have been identified and their number is increasing with the development of sequencing approaches (Tufan and Lachmann, 2020). The majority of 63 known pathogenic/likely pathogenic variants are located on exon 10 which encodes the B30.2/SPRY domain, responsible for the activation of caspase-1 (source: Infevers database, https://infevers.umai-montpellier.fr/web/search.php, last accessed 15 August 2024). The most common variant of *MEFV* is M694V (c.2080A > G), which is also a dominant variant in the Armenian population (Hayrapetyan et al., 2016). Other frequent exon 10 variants are M694I (36.0%), V726A (29.5%), M680I (26.2%), R761H (4.9%). These variants constitute almost 75% of all FMF patients and are associated with more severe disease courses among Mediterranean populations. Up to two-thirds of registered variants distributed over the entire gene are either not classified or classified as variants of uncertain significance (VUS) due to their unknown clinical association (Accetturo et al., 2020).

FMF is primarily an autosomal recessive disorder, however, this pattern is not uniform across all cases, adding complexity to genetic counseling and risk assessment (Manukyan and Aminov, 2016). Many individuals with FMF are compound heterozygotes. Different combinations of mutations exhibit a wide range of FMF phenotypes diverse in their symptoms and intensity, indicating incomplete penetrance (Procopio et al., 2018).

Clinical and research laboratories in Armenia use PCR/qPCR-based approaches to test the presence of the most prevalent 12–26 pathogenic mutations, such as FMF StripAssay (12 mutations, ViennaLabs, Austria) or FMF Multiplex real-time PCR kit (26 mutations SNP Biotechnology, Turkey). The common disadvantages of these kits are the limited scope of mutations tested (12 or 26 of known 63), risks of interference for closely located mutations (for example, M680I (G/C-A), I692del, M694V, K695R) in available qPCR tests, and limited capacity for further multiplexing. These limitations can be overcome with Sanger and next-generation sequencing (NGS). Sanger sequencing is considered a gold standard for accuracy and offers opportunities for studying genetic variability on the scale of an exon or a full gene (Schmidt et al., 2022). However, it has been proven that the costs of Sanger sequencing per sample are very high due to the small target range (∼1,000 nucleotides in a single run) limiting its use in clinical settings (at least in low- and middle-income countries (LMICs), such as Armenia). Furthermore, sequencing a single gene like *MEFV* using high-throughput Illumina NGS platforms is both technically challenging and costly. The installation and operational costs of these instruments require significant investments in infrastructure and a large number of samples is required per run to make it cost-effective. In contrast, nanopore sequencing (NS) developed by Oxford Nanopore Technologies (ONT) offers a more cost-effective solution, with capital and consumable costs as low as approximately $1,000, making it particularly suitable for use in settings with limited laboratory infrastructure. NS is actively explored for the identification of pathogenic variants in various genetic disorders, such as Familial Hypercholesterolemia (Soufi et al., 2022), tandem repeat disorders (Stevanovski et al., 2022), Werner Syndrome (Miller et al., 2021), sickle cell disease (Christopher et al., 2021), and others (Minervini et al., 2020; Oehler et al., 2023).

In this study, we focused on developing an affordable nanopore-based full-gene sequencing test for detecting *MEFV* mutations to assist in the diagnosis of FMF.

## 2 Materials and methods

### 2.1 Clinical samples

Fourteen patients (5 males and 9 females) were recruited from the Mikaelyan Institute of Surgery and Nairi Medical Center. FMF was diagnosed based on the classification criteria for autoinflammatory recurrent fevers (Gattorno et al., 2019) and molecular genetic analysis of *MEFV* mutations using a commercially available qPCR kit for the detection of 26 common FMF mutations (FMF Multiplex real-time PCR kit, SNP Biotechnology, Turkey). Two control non-FMF subjects (females) were also recruited from the Institute of Molecular Biology NAS RA. The project was approved by the Ethics Committee of the Institute of Molecular Biology NAS RA (IRB 00004079, Protocol N3 from 23.08.2021).

Five milliliters of morning fasting blood was collected in K_3_-EDTA tubes. Genomic DNA from 200 μL blood was isolated using QIAamp DNA Blood Mini Kit (QIAGEN, MD, United States), according to the manufacturer’s instructions.

### 2.2 Primer design and PCR amplification

We used the NCBI RefSeq (LRG_190) locus sequence of the *MEFV* gene (accession: NG_007871) for primer design. Five pairs of multiplex primers each targeting approximately 4 kb region of the *MEFV* gene were selected using the primalscheme tool for multiplex primer selection (Quick et al., 2017). Each primer pair generated overlapping amplicons, together spanning approximately 99% of the *MEFV* gene. This size was chosen to minimize the number of multiplex or singleplex PCR reactions and ensure that common long-range polymerase can effectively amplify the fragments.

Validation qPCR primers were designed to check the presence of all fragments in pooled PCR reactions. Each set of primers targets a region within the *MEFV* fragment. Primers for qPCR with intercalating dyes were designed using the IDT PrimerQuest tool (Integrated DNA Technologies, Belgium) (Supplementary Table S1).

Amplicons were generated using Q5 Hot Start High-Fidelity 2X Master Mix (New England Biolabs, United States), LongAmp Hot Start Taq 2X Master Mix (New England Biolabs, United States), and BioMaster LR HS-PCR (2x) Mix (Biolabmix, Russia). qPCR validation of the obtained PCR amplicons was performed using HOT FIREPol EvaGreen qPCR Mix Plus (no ROX) (Solis BioDyne, Estonia). PCR reaction mix and cycling conditions are provided in Supplementary Table S2.

The integrity of the amplicons generated using PCR was checked using 1.5% agarose gel electrophoresis for 30 min with 1 Kb Plus DNA Ladder (BioFact, Seoul, Korea). Amplified fragments were visualized using ethidium bromide. Amplicon quantification was performed with a Nanodrop spectrophotometer (Thermo Fisher, MA, United States).

### 2.3 Sequencing library preparation and sequencing

For each sample, generated amplicons were purified with a 1x ratio of AMPure XP beads (Beckman Coulter, Brea, CA, United States) to exclude small nonspecific fragments, equilibrated by mass and pooled. Sequencing libraries were prepared according to the manufacturer’s (Oxford Nanopore Technologies, Oxford, United Kingdom) protocol 1) using native barcoding kits (EXP-NBD104, EXP-NBD114) in combination with a ligation sequencing kit (SQK-LSK109) or 2) Native Barcoding Kit 24 V14 (SQK-NBD114.24). The final sequencing library amount was 20–50 fmol.

Sequencing was performed on the MinION 1 Mkb device using R9.4 Flow cells or Flongle flow cells and R10.4 Flongle flow cells. The average run duration was 12–18 h.

### 2.4 Sequencing data analysis and variant calling

Raw sequencing data was basecalled and filtered with guppy basecaller (version 6.0.1) with a “high accuracy model” parameter. Basecalled data was then demultiplexed for downstream analysis. The alignment was done with a minimap2 aligner (version 2.24-r1122) the ONT genomic reads option on the reference sequence of the *MEFV* gene (accession: NG_007871) downloaded from the RefSeq (LRG_190) NCBI database (Li, 2018). BAM files were sorted and indexed for further exploration in integrative genomic viewer and variant calling analysis using samtools 1.13 (Li et al., 2009). Variant calling of the aligned file was done with longshot 1.0.0 software for variant detection from error-prone reads (Edge and Bansal, 2019). Another tool used for variant calling was P.E.P.P.E.R. (r.08) which uses recurrent neural networks for variant detection from long read data (Shafin et al., 2021). The resulting variants were annotated with the Open Custom Ranked Analysis of Variants Toolkit (openCRAVAT) (Pagel et al., 2020). The presence/absence of a variant was further confirmed by examination of BAM files using Integrated Genome Browser v2.12.3.

### 2.5 Whole exome sequencing

Whole exome sequencing (WES) of the same DNA samples was performed by a commercial sequencing provider (Macrogen, South Korea). Libraries were prepared with Agilent SureSelect V5 (Agilent Technologies, CA, United States) whole exome sequencing library preparation kit. Sequencing was performed on the Illumina Novaseq 6000 instrument (Illumina, SD, United States). Variant calling was performed with the Genome Analysis Toolkit v4.3 based on published best practices (Van der Auwera et al., 2013).

### 2.6 Performance analysis

Accuracy, sensitivity, and specificity analysis were performed as described elsewhere (Bartol, 2015) using the caret R package. qPCR results on mutation genotypes were considered as ground truth.

## 3 Results

A total of 14 patients (5 males and 9 females) and 2 healthy controls (2 females) underwent full *MEFV* gene nanopore sequencing (NS). The DNA samples were sequenced with library preparation using R9 or R10 chemistry on both Flow cells and Flongle flow cells. Additionally, whole genome sequencing data generated on the Illumina platform was available for some of the samples (see Table 1).

**TABLE 1:** Combinations of sample preps and flow cells for nanopore sequencing of full-length *MEFV*.

Sample ID	SQK-LSK109 & EXP-NBD104 or EXP-NBD114	SQK-NBD114.24	Flow cell R.9.4	Flow cell R10.4	Flongle R9.4	Flongle R10.4	WES
F2	√				√		
F5	√	√			√	√	√
F8	√				√		
F10		√				√	√
F13	√	√	√			√	√
F15		√				√	√
F16		√				√	√
F19		√				√	
F20		√				√	√
F22		√				√	√
F24	√		√				
F27	√		√				√
F28	√		√				√
F31		√				√	
Ctrl1	√				√		
Ctrl2	√				√		

**FIGURE 1:**
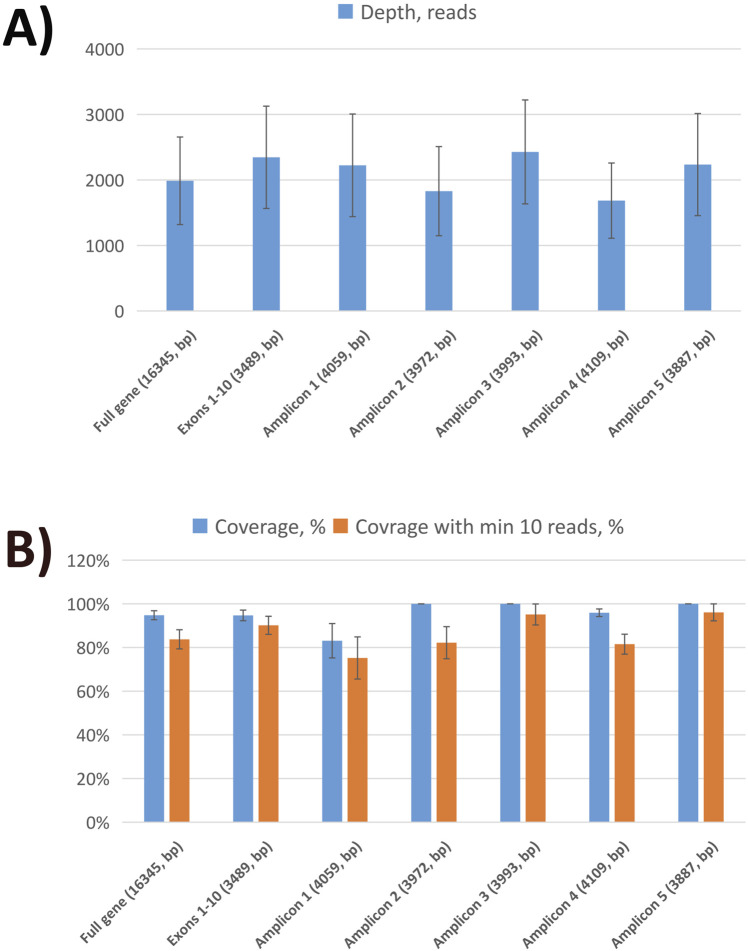
Sequencing statistics of *MEFV* gene using nanopore sequencing. **(A)** The average sequencing depth of full *MEFV* gene, exons, and individual amplicons. **(B)** Sequencing coverage of full *MEFV* gene, exons, and individual amplicons.

Nanopore sequencing statistics are shown in Figure 1. Using five tiling fragments, we achieved near-complete coverage of the full-length *MEFV* gene with balanced sequencing depth overall amplicons. The detailed statistics for individual runs are provided in Supplementary Data Sheet S1. The comparison of variants detected by NS and qPCR demonstrated a high level of concordance (Table 2). We performed a performance analysis of NS variant calling using qPCR results as the ground truth. Compared with qPCR the balanced accuracy of NS detection was 0.97 with a sensitivity of 0.93, specificity of 1.00, and F1 score of 0.97. From three NS replicates, two showed complete concordance of detected variants (samples F5 and F8). In one replicate of the F13 sample M694V was detected in a heterogeneous state (with V10 chemistry and R9.4 flow cell), while in the other replicate (with V14 chemistry and R10.4 flongle flow cell), the M694V variant was detected in a homozygous state concordant with qPCR results. Furthermore, in another sample (F19) additional pathogenic mutation V726A in a heterogeneous state was detected. Unfortunately, we did not have WES data for this sample to confirm the NS or qPCR results (Table 2).

**TABLE 2:** Comparison of variant detection of full-gene nanopore sequencing, qPCR (ground truth), and WES.

Sample ID	Patients ID	Chemistry	Flow cell type	Mutations (qPCR)	Mutations (nanopore)	Mutations (WES)
F02_MEFV	F2	R.9.4	Flongle	M694V (het)/ V726A (het)	M694V (het)/ V726A (het)	NA
F05_MEFV_R09_rep1	F5	R.9.4	Flongle	M694V (het)	M694V (het)	M694V (het)
F05_MEFV_R10_rep2	F5	R10.4	Flongle	M694V (het)	M694V (het)	M694V (het)
F08_MEFV_R09_rep1	F8	R.9.4	Flongle	M694V (het)/ V726A (het)	M694V (het)/ V726A (het)	NA
F08_MEFV_R09_rep2	F8	R.9.4	Flongle	M694V (het)/ V726A (het)	M694V (het)/ V726A (het)	NA
F10_MEFV_R10	F10	R10.4	Flongle	M694V (het)/ V726A (het)	M694V (het)	M694V (het)/ V726A (het)
F13_MEFV_R09_rep1	F13	R.9.4	Flow cell	M694V (hom)	M694V (het)	M694V (hom)
F13_MEFV_R10_rep2	F13	R10.4	Flongle	M694V (hom)	M694V (hom)	M694V (hom)
F15_MEFV_R10	F15	R10.4	Flongle	M694V (het)/ V726A (het)	M694V (het)/ V726A (het)	M694V (het)/ V726A (het)
F16_MEFV_R10	F16	R10.4	Flongle	M694V (het)/ V726A (het)	M694V (het)/ V726A (het)	M694V (het)/ V726A (het)
F19_MEFV_R10	F19	R10.4	Flongle	M694V (het)	M694V (het)/ V726A (het)	NA
F20_MEFV_R10	F20	R10.4	Flongle	M694V (het)/ R761H (het)	M694V (het)/ R761H (het)	M694V (het)/ R761H (het)
F22_MEFV_R10	F22	R10.4	Flongle	M680I (het)	M680I (het)	M680I (het)
F24_MEFV_R09	F24	R.9.4	Flow cell	M694V (het)	M694V (het)	NA
F27_MEFV_R09	F27	R.9.4	Flow cell	M694V (hom)	M694V (hom)	M694V (hom)
F28_MEFV_R09	F28	R.9.4	Flow cell	M694V (het)	M694V (het)	M694V (het)
F31_MEFV_R10	F31	R10.4	Flongle	M694V (het)/ M680I (het)	M694V (het)/ M680I (het)	NA
Cntrl1_MEFV_R09	Cntrl1	R.9.4	Flongle	-	-	NA
Cntrl2_MEFV_R09	Cntrl2	R.9.4	Flongle	-	-	NA

Moreover, both NS and WES showed additional variants outside the scope of the PCR mutation panel (Supplementary Data Sheet S2). For example, both WES and NS identified the R202Q (Supplementary Figure S2). In addition, 19 intronic, 5′- and 3′- UTR variants were discovered with NS (Supplementary Data Sheet S2).

## 4 Discussion

We present a protocol for full *MEFV* gene sequencing with accuracy comparable to qPCR for detecting common variants. Unlike qPCR, our method is not restricted to a predefined set of variants or locations and can discover additional clinically relevant variants that qPCR will miss. In contrast to WES, it enables the detection of variants not only in exons but also in intronic and regulatory regions. Although all variants observed in noncoding variants in the 3′UTR and intronic regions were previously annotated in ClinVar and classified as benign (see Supplementary Data Sheet S2) this also shows a possibility to identify pathogenic mutations located in non coding regions of the gene such as rs773992396 G>C located in intron 6 (Balta et al., 2020). Furthermore, the sequencing protocol enables more in-depth studies to elucidate the role of coding variants in disease. For instance, while the R202Q variant is classified as benign, recent studies have highlighted its potential association with an inflammatory FMF phenotype (Kandur et al., 2022). Our protocol has also proven effective with new sequencing chemistry and Flongle flow cells. The latter, especially when used with 12-sample multiplexing, makes full-length *MEFV* gene sequencing both cost- and time-efficient, with results obtainable within 12–24 h including bioinformatics analysis and report generation. Additionally, tree samples were analyzed in replicates, and the variant calling results were consistent across different nanopore sequencing chemistries, aligning with the qPCR genotyping results. In one sample (F13), the correct genotype was detected using the new V14 but not the V10 chemistry suggesting that switching to the latest flow cells and library preparation kits may enhance genotyping accuracy. These observations may point out that, in clinical settings, it may be beneficial to conduct replicates during the initial phase to gather sufficient data for assessing genotyping consistency and intra-sample variability before deciding to switch to genotyping based on a single sample. Furthermore, the R9.4 flow cells and associated sample preparation reagents were discontinued in 2023-2024, leaving V14 chemistry and R10.4 flow cells as the only available option. The cost of full *MEFV* gene sequencing for 12 samples on a Flongle flow cell is approximately 30 USD, which is comparable to, and often less expensive than, the 26-mutation qPCR panel commonly used in clinical labs in Armenia. With 1 Gb output (2.5 Gb theoretical output according to the manufacturer), 12-sample multiplexing can yield up to 6,000x sequencing depth per sample. The SQK-NBD114.24 kit supports up to 24-sample multiplexing, which could theoretically provide around 3,000x depth per sample - still adequate for variant detection. Additionally, the SQK-NBD114.96 library preparation kit allows for 96-sample multiplexing, making it a viable option for large-scale screening. However, with smaller sample flows, using 24- or 96-sample multiplexing may significantly increase turnaround time. Finally, compared to the Illumina sequencing platform, our protocol offers greater flexibility in the number of samples required for cost-effective multiplexing.

Our method significantly improves upon the protocol proposed by Schmidt et al. (2022), which uses 8 pairs of primers targeting only the exons of the gene, against only 5 primer pairs to target the full gene. The accuracy of variant calling can be further enhanced by switching the basecalling mode to “super accurate”. Although this would require more computational resources and time, it has been shown to outperform default basecalling settings (Ni et al., 2023).

The small sample size is the main limitation of our study. Furthermore, the amount of DNA samples was not sufficient to run NS for all samples in replicates. Nonetheless, our findings, along with previous results (Schmidt et al., 2022), warrant the applicability of full-gene nanopore sequencing of *MEFV* in clinical settings. Furthermore, even with this small sample, we were able to collect patients with diverse genotype combinations (heterozygotes for a single allele, compound heterozygotes, and homozygotes), which could mitigate potential sampling bias.

In conclusion, we present a cost-effective nanopore sequencing-based protocol for *MEFV* gene sequencing, making it particularly suitable for settings with limited laboratory infrastructure. It can enable widespread and accurate detection of FMF-related mutations, thus supporting public health initiatives aimed at controlling the prevalence of FMF, particularly in LMICs of endemic regions. This protocol could also lead to the discovery of novel pathogenic variants within the *MEFV* gene, expanding our knowledge of FMF genetics.

## Data Availability

The data presented in the study are deposited in the NCBI Sequence Read Archive repository, accession number SRP544892.
