# Supplementary material for: Genewise detection of variants in MEFV gene using nanopore sequencing
Source: Front Genet. 2024 Nov 29;15:1493295. doi: 10.3389/fgene.2024.1493295 (PMC11638185; doi:10.3389/fgene.2024.1493295)
Supplement: Supplementary file 2 [file Table1.docx]

***Supplementary Material***

# Supplementary Table 1. Primers used for the amplification of the full-length *MEFV* gene and for qPCR validation of amplified fragments.

| Fragment | Amplification primer sequence (5’-> 3’) | Fragment Length, bp | GC, % | qPCR validation primers (5’-> 3’) |
| --- | --- | --- | --- | --- |
| Amplicon  1 | F:CAAGTCCTTCATGTGGCTGGAA  R:AGTGCAAACTGGAAGAGGTGAC | ~4kb | 50% | F:GCGTCTGGCTGAGTTTCTTA  R:CCAAGGTGGGAGGATCATTT |
| Amplicon  2 | F: AAGAATGGAGAAGGGGAGCTCA  R:TCCTGACTCAGACTGCAGATGA | ~4kb | 50% | F: CTCGTGGCATGTCTGCTTAT  R:GGGCTAAAGACTGGAACTCATC |
| Amplicon  3 | F: GTGTAAGCAACTTGGGTTTGCC  R:CAAAGCAGGGTCTCAAGCAGAT | ~4kb | 50% | F: CCCAGTAGGACCACATCTTAAC  R:TACACGTTGAAGCCCTAACC |
| Amplicon  4 | F: ACCCCAGTACATGACAGTCTGT  R:CGCACCCAGCCTAAAACATGTA | ~4kb | 50% | F: CAGCCAGATCCCACATCTATAC  R:TGTGTTCTTCCCTCCATCAC |
| Amplicon  5 | F: GTGCCAGTCAGAATGGGAACTT  R:TCAGTACATGTCTTCACCCGGA | ~4kb | 50% | F: CAGCCAGATCCCACATCTATAC  R:TGTGTTCTTCCCTCCATCAC |

# Supplementary Table 2. PCR reaction mixes and amplification conditions.

| Component | Volume (μl) | Cycling Conditions |
| --- | --- | --- |
| Amplification PCR (Q5 master mix) | | |
| Q5 Hot Start High-Fidelity 2X Master Mix | 12.5 | Initial Denaturation 98ºC for 30sec  40 cycles at 98ºC for 10 sec  68ºC for 30 sec  72ºC for 2min  Final Extension 72ºC for 2 min  Hold 4ºC |
| Forward Primer (10μM) | 1.25 |  |
| Reverse Primer (10μM) | 1.25 |  |
| Genomic DNA (final concentration 20 ng) | 2-5 |  |
| Nuclease-free water | 8-5 |  |
| Total | 25 |  |
| Amplification PCR (LongAmp master mix) | | |
| LongAmp Hot Start Taq 2X Master Mix | 12.5 | Initial Denaturation 94ºC for 30 sec  30 cycles at 94ºC for 30 sec  59ºC for 1 min  65ºC for 3min20sec  Final Extension 65ºC for 10 min  Hold 10ºC. |
| Forward Primer (10μM) | 1 |  |
| Reverse Primer (10μM) | 1 |  |
| Genomic DNA (final concentration 20 ng) | 2-5 |  |
| Nuclease-free water | 8.5-5.5 |  |
| Total | 25 |  |
| Amplification PCR (LR master mix) | | |
| BioMaster LR HS-PCR (2x) Mix | 25 | Initial Denaturation 94ºC for 4 min  10 cycles at 94ºC for 10 sec  60ºC for 30 sec  68ºC for 2 min  30 cycles at 94ºC for 10 sec  60ºC for 30 sec  68ºC for 2 min  Final Extension 68ºC for 10 min  Hold 4ºC |
| Forward Primer (10μM) | 2 |  |
| Reverse Primer (10μM) | 2 |  |
| Genomic DNA (final concentration 20 ng) | 2-10 |  |
| Nuclease-free water | 19-11 |  |
| Total | 50 |  |
| Validation qPCR | | |
| HOT FIREPol EvaGreen qPCR Mix Plus (no ROX) | 4 | Initial Denaturation 95ºC for 12 minutes  45 cycles at 95ºC for 15 sec  60ºC for 30 sec  72ºC for 30 sec |
| Forward Primer | 0.5 |  |
| Reverse Primer | 0.5 |  |
| Amplicon (1-5) | 1 |  |
| Nuclease-free water | 14 |  |
| Total | 20 |  |
