# Supplementary material for: Genewise detection of variants in MEFV gene using nanopore sequencing
Source: Front Genet. 2024 Nov 29;15:1493295. doi: 10.3389/fgene.2024.1493295 (PMC11638185; doi:10.3389/fgene.2024.1493295)
Supplement: Supplementary file 3 [file Table3.docx]

***Supplementary Material***

| 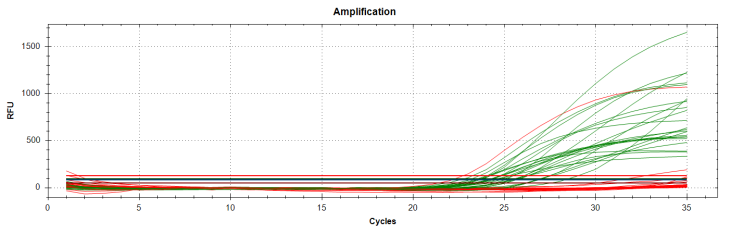  A | 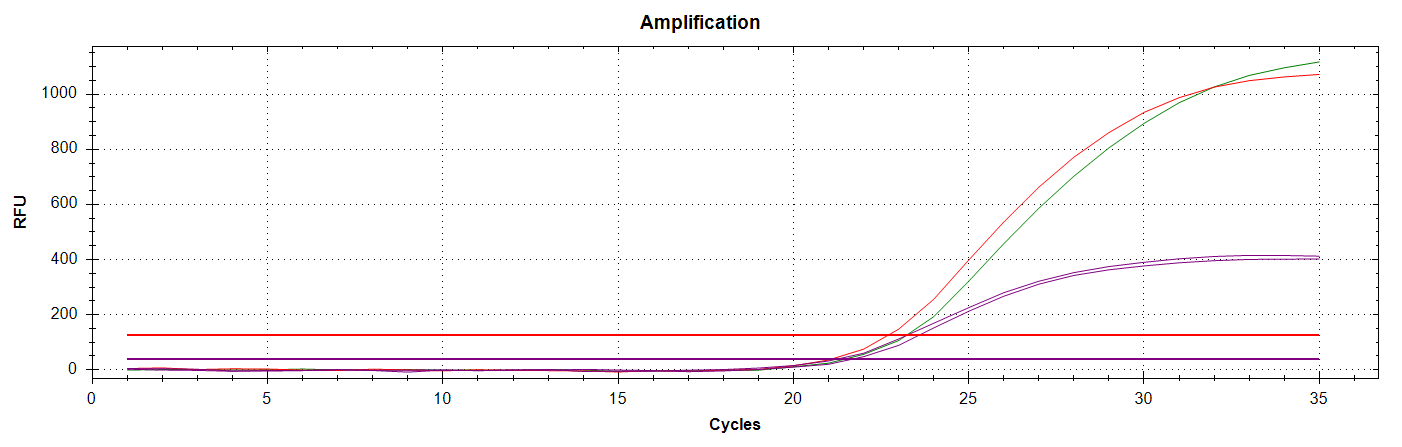  B |
| --- | --- |

**Supplementary Figure 1.** Exemplary result of qPCR genotyping of F5 sample with FMF Multiplex real-time PCR kit (26 mutations, SNP Biotechnology). Genotyping was performed in 12 PCR reactions. Green PCR curves denote wild-type alleles, red PCR curves denote mutant alleles, and violet PCR curves denote internal control. A) Overview of genotyping results. B) Identification of heterozygote genotype for M694V mutation.


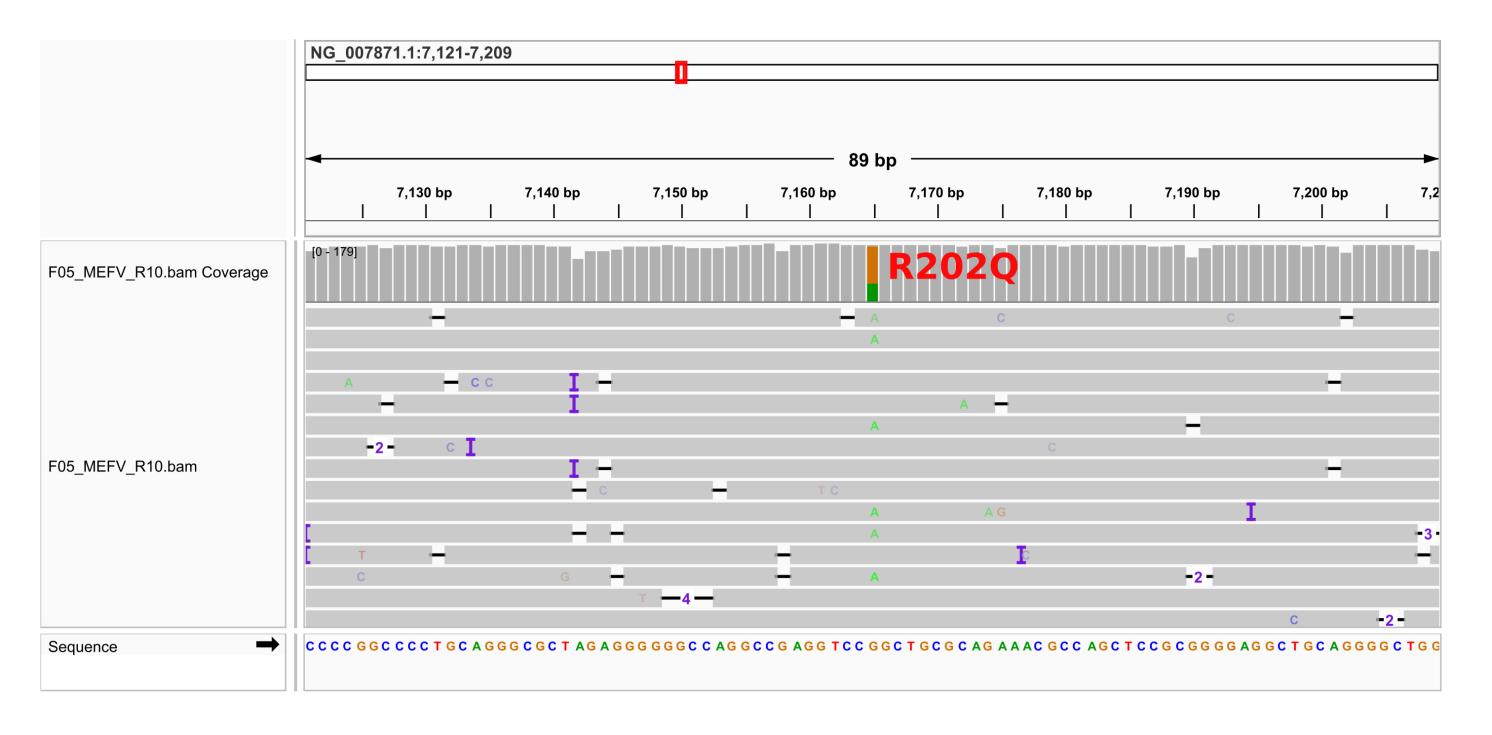


**Supplementary Figure 2.** IGV screenshot of R202Q mutation by nanopore sequencing in F5 sample. This mutation was also identified by WES.
